# A Call for a Socially Restorative Circular Economy: Waste Pickers in the Recycled Plastics Supply Chain

**DOI:** 10.1007/s43615-021-00056-7

**Published:** 2021-06-11

**Authors:** Anna Barford, Saffy Rose Ahmad

**Affiliations:** 1 University of Cambridge Institute for Sustainability Leadership, 1 Trumpington Street, Cambridge, CB2 1QA UK; 2 Murray Edwards College, University of Cambridge, Cambridge, CB3 0DF UK

**Keywords:** Informal sector, Waste management, Working poverty, Decent work, Global South

## Abstract

The labour-intensive task of waste collection for recycling is critical to contemporary forms of corporate circularity. In low- and middle-income countries, waste pickers underpin the recycling loop of the circular economy. Where informality and working poverty are the norm, waste pickers typically receive little social protection, work in dangerous conditions, and earn low wages. Nevertheless, waste pickers’ work addresses multiscalar environmental problems from localised flooding of plastic-clogged waterways, to preventing the release of greenhouse gases when plastic is burnt. Here, we review recent academic and grey literature on waste picking, the social circular economy, and corporate circularity to understand the role and position of waste pickers in the contemporary circular economy. We explain how given the recent outcry against plastic waste, and subsequent corporate commitments to plastic recycling, there has been greater action on material flows than in support of the people who move these flows. Overall, the corporate response remains limited, with a general preference for recycling over redesign and only a fifth of packaging accounted for. Based on this review, we present two models. The first is a hierarchy of plastic recycling showing the foundational role of waste pickers in the recycled plastics supply chain. As plastics move up the hierarchy, their value increases and working conditions improve. We also propose a new model for a socially restorative circular economy which provides fair pay, safe working conditions, social protection, legal rights, voice, respect, services, and education. Some governments, co-operatives, non-governmental organisations, and businesses are already working towards this—and their work offers pathways towards a new standard of fair trade recycled materials. We argue that for true sustainability and the best version of circularity to be achieved, deeply ingrained social challenges must be resolved.

## Introduction

Recent moves to recycle our way out of the plastics problem must include attention to the waste workers making this possible. Informal waste workers have long sifted through the rubbish of others, extracting value where possible. This scenario, played out across most continents, reflects the imbalances of the wastefulness of the haves, and the need to make a living of the have nots. Public outrage with plastic pollution crystallised in 2017 following the British Broadcasting Corporation (BBC) documentary Blue Planet II, narrated by Sir David Attenborough, which built upon earlier scientific and journalistic work to publicise the extent of the plastics problem [22]. Since then, several major companies have issued bold statements committing to substantial increases in the recycled content of their products or packaging. These companies include manufacturers such as the computer hardware company Dell, chemicals giant Dow, and the consumer goods manufacturers Unilever and Nestlé (Table 1). Many governments also reacted. For instance, in 2017, the Kenyan government banned plastic bags, in 2018, China banned the import of plastic waste, and in the same year, the UK government established a £20 million Plastics Research and Innovation Fund [41]. At the international level, the United Nations Environment Assembly in Nairobi, March 2019, saw 170 countries pledge to adopt sustainable production and consumption patterns [91]. Without intervention, business as usual would put us on track for ocean plastic to outweigh fish by 2050 [103]. In low- and middle-income countries, many of the interventions to retrieve plastics from the environment rely heavily upon the cheap and insecure labour of a sizable informal workforce.

Despite the imperfections of this version of circularity, waste collection by informal waste pickers is critical to the recycling loop of the circular economy in many countries. One well-established criticism of recycling is that it is the least profitable and least resource efficient loop in a circular economy [68, 86]. Furthermore, recycling goals can distract efforts from bolder versions of the circular economy [97]. Preferable options include reusing existing plastic items and sharply reducing the production of new plastics. Nevertheless, while slow to make more substantial changes to their modes of operation, businesses have been relatively quick to adopt and extend the practices of recycling and resource reduction (Moulis, unpublished), and in Europe recycling is central to around half of industrial circular mitigation ([16], p. 45). We were unable to find the equivalent figure for other regions where circularity is lower on the agenda. Waste picking provides an income for tens of thousands or even hundreds of thousands of people in many countries where opportunities for good-quality work are scarce [4, 8, 9]. However, this work is tarnished by low pay, lack of social or legal protection, and poor safety standards ([55]; Table 2). There is a strong dependence upon waste pickers in current modes of circularity, and waste pickers themselves rely on this work to get by. With the global ‘circularity gap’ hovering at 91.4% [15], and decades of work needed for a major transition [68], the circular economy is still being built and social priorities can be designed in at this stage. This is preferable to retrofitting social concerns to an already existing circular economy, with all the lost opportunities that would entail.

**Table 2:** In informal waste collecting work in four countries (originally presented by [4])

	Informal waste collectors	Waste picker contribution to recycling	Working conditions and challenges	Demographics	Data sources
Brazil	380,000 (in 2008, refers to all waste workers)	90% (of collected recyclable packaging is from waste pickers)	Lack of equipment, often earn under the minimum wage, seasonal variations in prices, exploitation	40% illiteracy or incomplete primary education, some workers are homeless	From review by [76]
Indonesia	2915 in Bandung (>1/1000) 40,000 in Jakarta in 1992	86% for paper 8% for plastics (contribution to what is recycled in Bandung)	Health hazards from medical waste; no potable water where waste pickers live (in Bantar Gebang)	Predominantly young people (20s–30s), also children. 99% of unpaid workers are women (in Bantar Gebang)	[83, 79]
Nigeria	1,000,000 (urban Nigeria)	80% (of all Lagos recycling is informal)	Some enjoy the flexibility of not having a boss; sometimes incomes exceed the minimum wage	Low levels of education, low social status, and poor living conditions	[58, 67]
South Africa	60,000–90,000	80–90% (of all paper waste and packaging)	Women earn less than men	Lack social protection, crowded living quarters with poor hygiene	[33, 60, 78]

Unlike the environmental and resource management potential of circularity, the social mechanisms and benefits remain under researched [14, 34, 42, 87]. There has been a tendency to assume that economic benefits will automatically bring environmental and social benefits [94], though this assumption is now being confronted with calls for further research into the wider influences the circular economy has on people, society, and climate change [43, 68]. An *amended circular economy* argues for a redefinition of the circular economy that acknowledges the social and political dimensions to the concept [38]. Our aims here are to contribute to this amendment process, and to make sense of the role, circumstances, and interdependence of waste pickers in the circular economy. We also reflect on why plastics are now being addressed while the social dimensions are largely overlooked in government and corporate responses. Plastic pollution is often described as a wicked problem which connotes a degree of complexity that can paralyse effective responses [84, 89], but nevertheless plastic pollution receives considerable attention and some coordinated responses. So why do the waste pickers who handle this material receive such scant attention in circular economy discussions?

To address these points, we focus on the role of labour within today’s incomplete and imperfect circular flows, highlighting the low quality of work available to waste pickers within the recycled plastics supply chain. To begin, we briefly outline our methodology, before considering theoretical approaches which position the circular economy within wider society. Secondly, we address the need and potential for job creation in the circular economy. Thirdly, we emphasise that while the term *circular economy* has a recent currency, circular practices have long been used to meet a combination of resource (including financial) and local environmental needs, for example the practices of waste pickers. New circular models often build upon these pre-existing networks and working norms. Fourthly, we elaborate on who ‘waste pickers’ are and the nature of their livelihoods. Fifth, we demonstrate how recent commitments to increased circularity of plastics have heightened corporate reliance on waste pickers. Given this reinvigorated reliance on waste pickers, the paper concludes with a socially regenerative model for engaging waste pickers, which revalues the people involved in processing circular material flows.

## Literature Review

A desk-based literature review of relevant literature is used to assess theoretical and practical research into the recycled plastics supply chain. The search engine ‘Google Scholar’ was used to identify relevant papers, using search terms including: ‘circular economy’, ‘post-consumer recycled plastics’, ‘informal solid waste management’, ‘waste pickers’, ‘social circular economy’, ‘decent work and the circular economy’, and ‘circular economy job creation’. The bibliographies of the initial papers were used to identify further literature. Studies from Africa, Asia, South America, and Europe were included, with most papers published within the past decade, and especially the years 2017–2020. The recycling and post-consumer commitments of five multinational corporations were also reviewed, drawing upon their annual reports and press releases. The corporations (Dell, Dow, Hewlett Packard, Nestlé, and Unilever) were selected for their stated public commitments to a circular economy transition. The aim was to understand how corporations are enacting the circular economy. The review evidenced the relatively sparse literature on recycled plastics in lower income countries, in contrast to literature on China and Europe, and how people are largely overlooked in mainstream approaches to the circular economy. This corroborates Kirchherr et al.’s [48] systematic analysis of 114 circular economy definitions in scholarly and practitioner discourse, which found only 18–20% of circular economy definitions referred to ‘social equity’. This paper responds to the need to focus on both lower income nations and the social dimensions of circularity.

## Theoretical Approaches to Circularity in Society

While there is limited circular economy research focused on the social, several approaches have been adopted to bring people and society into circular economy thinking, and those that do acknowledge this oversight have called for more work to address this gap (e.g. [61, 63, 80, 95, 96]). With specific reference to waste work, Costas Velis [95] measures the informal recycling sector against the Sustainable Development Goals (SDGs). Emphasising the gulf between these goals and reality, the United Nations Educational, Scientific and Cultural Organisation reported how some waste pickers consider themselves to be in a social category associated with ‘sub-human characteristics’ [64]. Rigorous discussions and tangible evidence of a socially orientated circular economy, especially for waste pickers, remain scarce. Nevertheless, the social and solidarity economy and *amended circular economy* offer theoretical lenses for a more inclusive approach to the roles and positions of people within the circular economy.

Social and solidarity economy thinking is proposed as a route to bring people into discussions on the circular economy. This approach promotes reciprocity with an interest in community, social, and environmental goals, rather than reciprocity out of necessity or built upon unequal social relations [61]. At its core is a commitment to ‘the primacy of people and work over capital in the distribution of revenues’ ([17], 16). Given how the economy is embedded within the social realm (Polanyi, 1994 in [61]), the success of the economy rests upon that of society. Furthermore, a successful economy should meet both social and environmental needs [75]. Towards this goal, social and solidarity economy approaches promote democratic participation in economic activities as well as fair trade programmes for more equitable labour conditions. With respect to the circular economy, Moreau et al. *[*61*]* contend that conditions to support more solidarity-based production and consumption systems could lead to more resource-efficient activities. This can work by increasing labour-intensive activities while raising the quality and diversity of human work involved in remanufacturing and recycling.

**Fig. 1:**
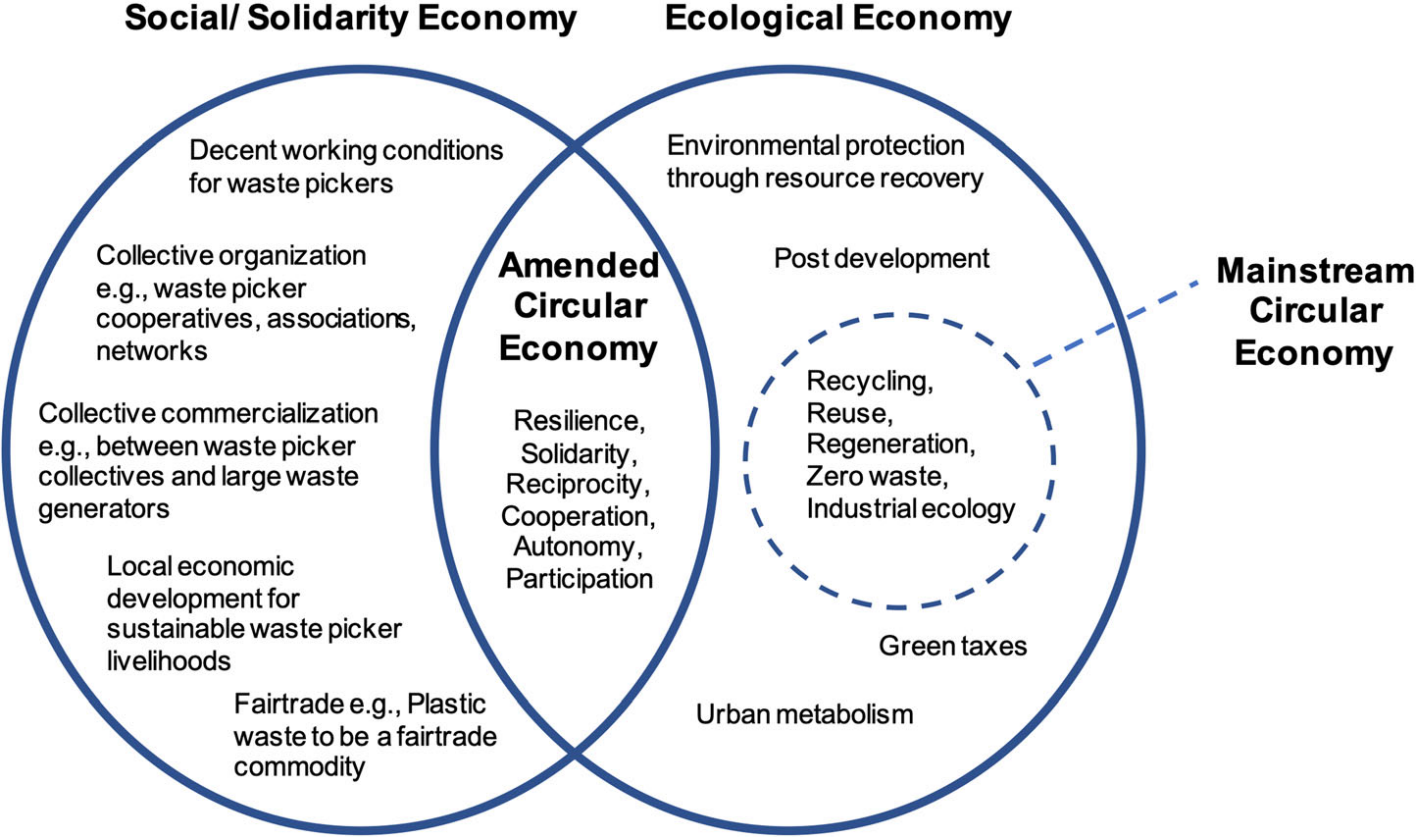
The amended circular economy for the Global South. Based upon [38] (p. 10), and modified with reference to the work of waste pickers

Mainstream circular economy thinking focuses tightly upon material flows, to the exclusion of their wider context. This has allowed for much needed thinking on how to design and improve material flows. The *amended circular economy* connects the circular economy to its wider societal and political context [38]. Amending the circular economy goes beyond simply inserting the social into circular economy formulations, but demands a redefinition of the circular economy by incorporating elements of the social and solidarity economy and the ecological economy (*ibid.*, Fig. 1) in addition to the existing contributions from thinking on industrial ecology, cradle-to-cradle, and biomimicry. Ecological economy emphasises precaution, responsibility, and the interdependence between human economy and ecosystems [28, 37]. Coupling the ecological economy with the social and solidarity economy described above draws attention to innovative forms of economic interactions, where people and the environment transcend profit generation and efficiency orientation. The *amended circular economy* offers an equity-oriented framework in response to the capitalistic business models that circular economy approaches currently emphasise, underscoring the importance of social practices and everyday experiences to create a more complete circular economy. With reference to waste management, Gutberlet et al. [38] propose that insights and interventions developed by informal and organised waste picker groups are proactively incorporated into a more participatory and inclusive approach to circularity.

The framework of an *amended circular economy* adds balance to the narrow focus on materials in mainstream circular economy thinking, and highlights the wider social and political contexts which also shape material flows. It also offers tools to understand the intersection of environmental and social imperatives, which we understand to be fundamentally interdependent. We build upon this approach to address the social and political context of recycling loops in lower income countries, developing an explanation as to why social concerns lag behind *environmental* protection. We then use the principles of a regenerative circular economy to develop a socially restorative model for waste pickers. While the *amended circular economy* approach works with grassroots innovations in waste management, we engage more with dominant modes of neo-liberal capitalism which drive much of the discussion about circular transitions. We advocate targeting ten specific social challenges by a variety of stakeholders, and support a transition to fair trade recycled plastic, to improve waste pickers’ livelihoods, repositioning them as integral to a circular transition.

## Work in the Circular Economy

There is great need and potential for job creation in the circular economy. By 2030, it is thought that a possible net total of 7–8 million new jobs could be created in the circular economy, with a further 50 million jobs reallocated from linear to circular processes [47]. This net job creation is partly due to the circular economy demanding more labour than the linear economy, as manufacturing and repairing goods are more labour intensive than resource extraction [86]. A shift towards the more labour-intensive activities of remanufacture and repair is likely to increase labour demand. However, this projection of new jobs comes with caveats. Firstly, these future jobs require the widespread roll-out of skills training. There is a risk that without the right training, and essential political support, far fewer jobs will be created which could worsen the global shortage of work. Secondly, those losing their jobs and those gaining a new job will not always be the same people, with people working in the extractive industries expected to be disproportionately impacted by job losses [47]. There is likely also to be a new distribution of jobs, both in terms of geography and sector of the economy.

We currently face a dual crisis of insufficient work and poor-quality work [8, 9]. Thus, discussions of new jobs require attention to their quality, and fair pay is one marker of the quality of a job. In Sub-Saharan Africa, c.70% of young people are in working poverty, and the equivalent number for South Asia is c.55% [45]. In assessing the social contribution and impact of the circular economy, it is important to acknowledge existing human crises including widespread poverty and premature death from curable diseases [7, 20], in addition to weak labour market demand in many places, which set the context in which circular economy models are being developed. As the circular economy approach aims to improve current modes of production, it makes sense to integrate discussions not just on the need for work, but also on the need for that work to be decent. We maintain that ‘Our unfavourable starting point places a sizable burden upon new models of circularity to solve the multiple and interlocking challenges that are not of its making.’ ([4], p. 2). There is a serious risk that a circular transition could entrench societal cleavages still further if the social elements are not fully addressed in planning, rollout, and reporting. Just as new modes of production can be used to sidestep further environmental destruction, they could also rework persistent social challenges. The need to get this right cannot be understated, and achieving this would contribute to the Sustainable Development Goals (SDGs). In particular, a socially regenerative circular economy can contribute towards SDGs on poverty (SDG1), hunger (SDG2), gender equality (SGD5), decent work (SDG8), inequality (SDG10), in addition to that concerning responsible consumption and production (SDG12) which is more intuitively aligned to the circular economy.

Our broad definition of work in the circular economy extends to include unpaid labour in the home [8, 57], alongside paid work outside of the home. Unpaid domestic work (often categorised under the more general term ‘consumer behaviour’) within the circular economy includes activities such as repairing and maintaining items, sharing, sourcing second-hand items, and sorting waste. These tasks tend to fall disproportionality upon women, and on average, women spend 3.2 times more time on domestic and care work than men do, globally [46]. Amongst the many studies of the circular economy, consumers have been somewhat overlooked [42], and more attention has been paid to circular economy work outside of the home. This includes paid work in distribution, collection, repair, and remanufacture [47], as well as the labour required for systems redesign and change [90]. In Slovenia, for example, green policies resulted in increased farm labour demand in response, to drive the reconfiguration of systems and realignment of practices (*ibid.*). Some newly created jobs will be transition-specific, to design and build new systems and processes. Other forms of work will continue. Without major reforms, some of this work will be paid, some even celebrated, some invisible, and some paid little or nothing at all.

While idealised forms of circularity are hard to find in reality, many are already working towards greater circularity by narrowing loops (decreasing material and energy use per product), closing loops (ensuring both production material and the finished product can be recycled), and slowing loops (creating durable and long-lasting products to slow consumption) [10]. Currently, waste collection is central to the recycling loop of the contemporary circular economy, yet it is poorly paid, informal, insecure, and often dangerous [36, 76, 95]. Despite the many local and national interventions aimed at improving the lives and livelihoods of waste pickers, there is still a long way to go ([30, 37, 70]; [12, 59]; [52, 65]). Overall, the recycling loop of the circular economy continues to fail according to social indicators for waste pickers.

## Informal Waste Picking in Context

With only a decade of dedicated thinking and advocacy behind it, ‘circular economy’ is still a relatively new term. It is widely acknowledged that many of the practices and processes which fall under the remit of ‘circular economy’ were forerunners of this new parent concept. This includes the push to reduce, reuse, and recycle; a history of repair, reuse, and at times rationing of consumption. These pre-existing practices were already contributing to many of the same resource and environmental goals which circular economy models now target. Circular economy goals build upon these existing structures and processes, tapping into, supporting, enhancing, and sometimes rewiring existing flows. Although it is important not to glorify poverty, resource scarcity leading to careful resource management is central to circular economy thinking. As such, it is inevitable that a certain amount of circularity plays out in low-income settings, albeit not automatically labelled as such.

Frugal innovation using waste to make a living in resource poor settings brings a value to waste not just for its reuse potential, but also as an income source. *Jugaad*, a Hindi word with its roots in the Punjabi word for a makeshift vehicle, refers to ‘the gutsy art of spotting opportunities in the most adverse circumstances and resourcefully improvising solutions using simple means.’ ([73], p. 4). This approach, ubiquitous in India and beyond, involves actions as simple and intuitive as reusing food packaging for food storage to building new items by cobbling together the old (*ibid.*). Motivated by the need for an income, and at times also seeking to address an environmental problem, selling sorted waste is an income source for an estimated 1% of the urban labour force in developing countries (Women in Informal Employment: Globalising and Organising [WIEGO], no date). While the need to make a living in a context of restricted job opportunities pushes many waste pickers into this line of work, others seek to solve the problems created by this waste, as was the case for Isatou Ceesay of N’jau village in the Gambia. She observed direct damage from plastics, which sometimes killed the goats who ate it, reduced soil fertility, and when burnt released toxic gases—she later learned to crochet and created jobs and income streams for people crocheting plastic bags into useful items [23]. This story embodies some ideals of local leadership, job creation, and the improved management of plastic waste.

**Fig. 2:**
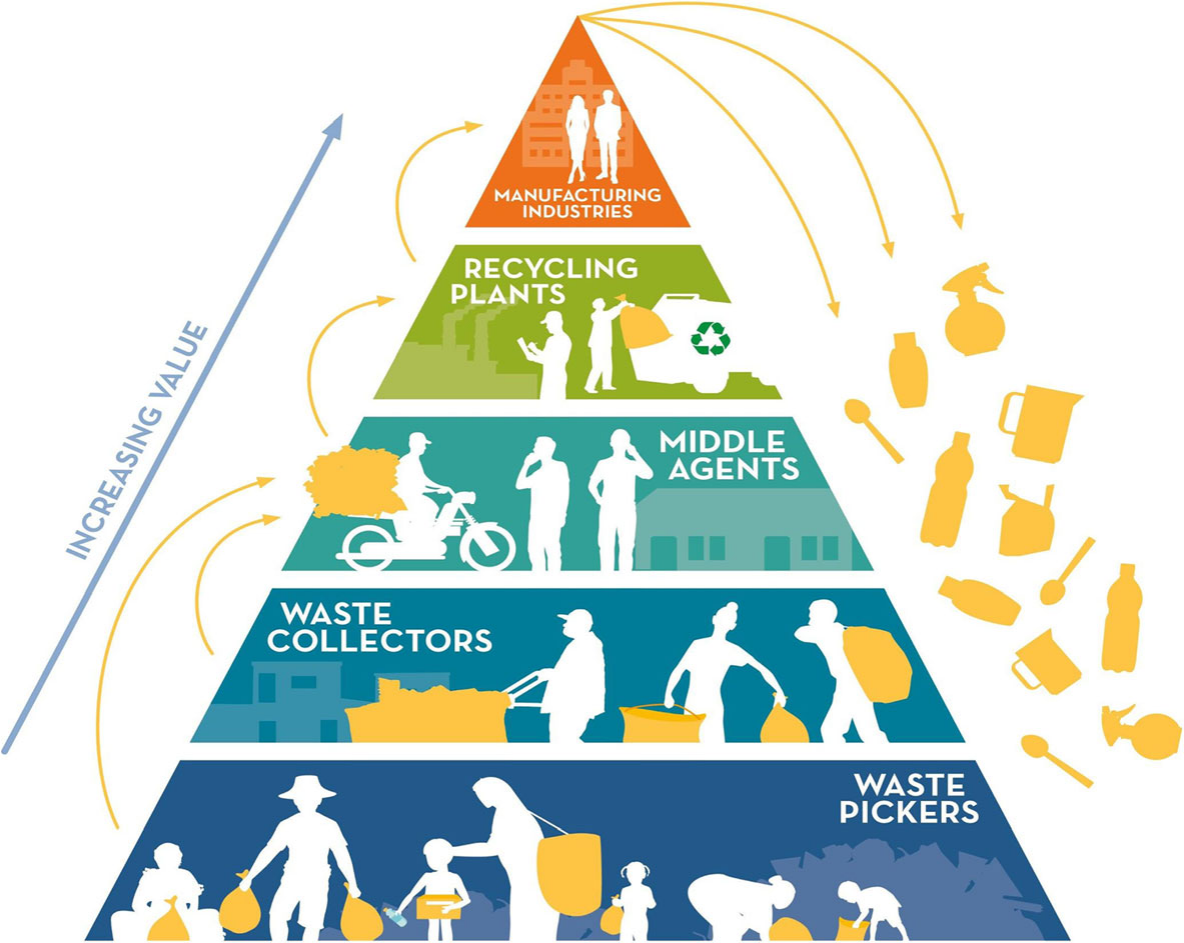
Hierarchy of plastic recycling. Reminiscent of the ‘Pyramid of Capitalist System’ diagram (Lockhoff, 1901), the Recycling Labour vs Recycling profit pyramid [98], and figures featured in Wilson et al. [101], Hayami et al. [39], and Schenck and Blaauw [81]. This diagram was designed with reference to a series of studies detailing the flow of recyclable plastics in Colombia (Medina, 2008; [64]), India [39, 64], Indonesia [64], Kenya [30], and South Africa (Mkhize, 2020; [81]). Note that this general model varies with local recycling infrastructure and institutional context. Much waste remains uncollected and unmanaged

Waste pickers have long-engaged in resource recovery, retrieving materials from waste streams and redirecting them into the recycling or reuse economy, making a living while also minimising resource loss. Waste picking preceded the conceptual framing of the circular economy; hence, arguably, waste pickers have been at the heart of the circular economy since its inception [30, 37, 82]. New circular economy recycling schemes have built upon this livelihood form, creating new work based upon increased demand for recyclable materials. However, it is not well established who is responsible for such poor-quality work existing. Firstly, informal poorly paid work is widespread in low- and middle-income countries, and preceded moves towards circularity, so there is a diminished sense of causality or immediate responsibility. Secondly, while waste pickers are well connected by hierarchical waste flows, the connection to more formal structures is often mediated by middle agents. The indirectness of this connection interrupts contact and engagement which can diminish a sense of responsibility to act ([3, 5], Fig. 2). Thirdly, despite high profile campaigns against poor living and working conditions, including Make Poverty History, Occupy, as well as the officially sanctioned Millennium and Sustainable Development Goals, there is minimal demand for labour improvements in the recycled plastics supply chain from the media, public, or governments. This contrasts with plastic materials for which responsibility seems easier to trace—given how plastics are introduced to the environment, often stamped with corporate branding which unambiguously marks connection and responsibility.

Formal recycling infrastructure and regulation are more recent for many low- and middle-income countries, and many still have inadequate infrastructure to manage the volumes of waste accumulation. A historical review of recycling in South Africa, one of the richest and yet most unequal economies on the African continent, shows considerable progress in recycling since 1990 [102]. This study maps out five stages moving from landfilling, to emergent recycling, then increased regulation, followed by a drive for Extended Producer Responsibility, through to the present with South Africa entering a circular economy stage [32]. This increasing formality often relies upon formal and informal waste pickers. Andreas [2] highlights how the legal and illegal, and the licit and illicit are intertwined; the same can be said for formal and informal dimensions of the recycling supply chain which in many settings are co-dependent. As waste pickers are the foundations of the recycling circular economy in many low- and middle-income countries, we next elaborate upon the demographics and working lives of this occupational group.

## Waste Pickers

*‘In developing countries, around 1 per cent of the urban workforce is engaged in recycling: collecting, recovering, sorting, grading, cleaning, baling, or compacting waste, as well as processing waste into new products. … The ILO estimates that 15-20 million persons worldwide earn their living from recycling waste.” (WIEGO, no date, no page)*

With tens of millions of people involved in waste picking, and in light of the *amended circular economy*’s call to engage with the insights of informal and organised waste pickers (see [38]), we now unpack the term waste picker to better understand who is in this group and what this work entails. Contrary to the common assumption that workers in the informal sector are disorganised or homogenous, waste picking work is organised and highly structured (Fig. 2; [1, 55, 79]), and collectively waste pickers process the vast majority of recyclable waste where they work (Table 2). *Waste picker* was adopted as a dignified English language name for this group of workers at the first World Conference of Waste Pickers held in Bogotá, Colombia; the name selected in Spanish was *recicladores* [12]. Waste pickers are usually unrecognised, informal, and in India their ‘sheer number’ and ‘scattered geographies’ can render them almost invisible ([74], p. 181). As a diverse group, working in many countries, there are nevertheless some broad similarities in the challenges faced.

One widespread characteristic of waste picking is that it is often seen to be low status, and is usually done by the more marginal members of a society. For example, in Brazil, amongst waste pickers, illiteracy levels are around 40% [76]. In both Pretoria, South Africa, and Delhi, India, most waste pickers are rural migrants to the city [39, 81]. In India, waste pickers are often Dalits, people positioned at the bottom of the hierarchical caste system and assigned to low status occupations [51]; thus, occupation reinforces low social status [64]. Furthermore, in Muslim majority countries, there is a tendency for non-Muslims to take on the impure task of waste work (Medina, 2002 in [64]). A South African study showed a clear racial dimension as all waste pickers surveyed were black [81]. Within groups of people who work with waste, the better paid, higher status roles tend to go to those who are more empowered and there is a gendered dimension to this. For instance in Durban, South Africa, men tend to collect more valuable materials such as metals, whereas women collect lower value materials including cardboard, paper, and glass; men are able to employ other workers whereas women rely on the unpaid work of others in their household (Mkhize, 2020). In a study in Bantar Gebang in Indonesia, 99% of waste pickers who were unpaid were female [79]. The low status of waste picking and the sub-divisions within it reinforce identity-based forms of discrimination as well as socio-economic disadvantage.

The precarity of waste pickers is captured by the South African terms for this work—‘skarreling’ and ‘minza’—which translate as ‘trying to survive’ [81]. The challenges which confront waste pickers intersect with one another to further increase levels of vulnerability. Perhaps at the most basic, waste pickers are often paid below the minimum wage for their country [58, 67, 76]. This low pay is made worse by a lack of a wider social safety net, or social protection for times when work is disrupted, including during the global financial crisis and COVID-19 pandemic [33, 60, 77, 78]. Waste picker Beauty Ncube describes the impact of COVID-19 lockdowns on her family: ‘I was providing for my kids with the little money I'm getting, but now, I'm starving.’ (in [50]). Waste pickers often work in unsafe conditions handling hazardous materials without protective equipment, and live in crowded quarters without access to clean water or good hygiene [33, 58, 60, 67, 76, 78, 99]. All this amounts to a system which depletes the workers who drive it forwards. Yet, growing recognition of waste pickers’ contribution to the circular economy has led many major businesses to engage them to achieve circular economy goals.

## Corporate Commitments to Circularity

Recent commitments to increased circularity of plastics have heightened corporate reliance on waste pickers. Circular policies and practices are most vigorously promoted in higher income nations, with leading initiatives found in the EU, Canada, and China [49]. However, the reach of multinational corporations with their global supply chains and distribution networks has created enormous international flows of raw materials and manufactured products, generating large amounts of waste. For example, while multinational corporations may not manufacture products in all locations, their effective distribution systems direct durable packaging into the markets, streets, rivers, oceans, and even the air of some of the poorest communities. Coca-Cola, PepsiCo, Nestlé, and Unilever are thought to be responsible for more than half a million tonnes of plastic pollution in India, the Philippines, Brazil, Mexico, Nigeria, and China [88]. These globalised flows of the dominant linear economy mean that the circular economy is highly relevant to low- and middle-income countries, as both a reactive and proactive approach to managing plastic waste, while holding the potential to generate additional benefits for local communities [38].

Resource recovery through global recycling networks is currently a key avenue for corporate circular economy work, distracting attention from more disruptive and efficient versions of circularity [35, 97]. This avenue has been accelerated by the recycling commitments of several leading multinational companies who have adopted goals of greater responsibility for their plastic pollution. Some key commitments from multinational companies in the fast-moving consumer goods, chemicals, and electronics sectors are listed in Table 1. For many companies, these commitments are integral to transforming business models, which is significant as circular business models are widely recognised as representing the core of the circular economy [53]. These specific commitments demonstrate attention to the social, in addition to the need for circularity (Table 1). For example, the approaches deployed to achieve the stated commitments often involve corporate engagement with low- and middle-income countries, whereby partnerships are brokered with organisations, community groups, and individuals who engage in waste picking and recycling activities. Such enhanced relations have begun to generate tangible advantages for some waste pickers.

**Table 1:** Companies’ self-reported waste management goals, approaches, and successes. Sources: Dell (2017), Dow (2019), Nat Geo (2020), HP [44], Nestlé [66], and Unilever [92]

Company	Commitment	Approach
Dell	*All packaging will be made from recycled or renewable materials, by 2030.*	Global Take-back scheme in 83 countries enables disposal of old products, for repair then reuse. Partnerships used to manage PET and HDPE. E.g. Dell co-founded NextWave Plastics with Lonely Whale to manage ocean-bound plastics. In 2019, 66,635 lbs (30,225 kg) of ocean-bound plastic used in new Dell-branded packaging. Aim to recover and recycle >3 million lbs of marine plastic over 5 years.
Dow	*All packaging to be reusable or recyclable, by 2035.* *Collect, reuse or recycle 1 million metric tonnes of plastic through direct actions and partnerships, by 2030.*	Created an Impact Fund to tackle poor waste management through education, clean-up and innovation working with NGOs to connect buy-back centres, sorting facilities, collectors and recyclers. E.g. Dow’s ‘Recycling for a Change’ project, São Paulo: with NGOs Fundación Avina and Boomera bring training and equipment to five waste picker cooperatives. Increased productivity by 70%, sales by 50%, salaries raised above minimum wage, improved quality of post-consumer plastic resin.
Hewlett Packard	*Increase recycled content in products to 30%, by 2025.*	Sources recycled plastic for a circular supply chain, particularly locally-sourced ocean-bound plastic. E.g. Turning Off the Tap Haiti Project with First Mile Coalition: transferred closed-loop manufacturing knowledge. By September 2019, >1 million lbs (>453,592 kgs) of ocean-bound plastic were sourced for new HP products; 1100 x 1 month jobs were created in Haiti.
Nestlé	*All packaging will be recyclable or reusable, by 2025.* *Reduce use of virgin plastics by one third, by 2025.*	Design and implementation of Extended Producer Responsibility schemes to improve recycling rates and infrastructure in 20 countries, accounting for >50% of their plastic usage. E.g. 3-year partnership with Project STOP, East Java, 2019: contributed EUR 1.5 million towards local research and training, built waste-collecting and sorting infrastructure.
Unilever	*Increase post-consumer recycled plastic material in packaging to at least 25%, by 2025.* *Help collect and process more plastic packaging than they sell, by 2025.*	Investment and partnerships in the collection and processing of 600,000 tonnes of plastic annually. E.g. Partners with Mr Green Africa in Kenya to engage often-exploited ‘pickers’. Created 2700 new waste picking jobs.

Dow’s commitment to ‘Stop the Waste’—by enabling a million tonnes of plastic to be collected, reused or recycled—has been advanced through the ‘Recycling for a Change’ project in São Paulo, Brazil. This project supplies plastic for the production of post-consumer plastic resin, with the Non-Governmental Organisation (NGO) Fundación Avina working to improve training, equipment, and professionalism for waste pickers [21]. Similarly, in an effort to reach their commitment to increasing the use of post-consumer recycled plastic material in its packaging to at least 25% by 2025, Unilever’s investment in circularity and partnerships has facilitated the collection and processing of 600,000 tonnes of plastic annually [92]. Their partnership with a for-profit organisation, Mr Green Africa in Kenya, has been integral to the reported creation of jobs for 2700 waste pickers [92]. Such commitments to recycled materials by those companies who have a vast influence on the global flow of resources therefore hold potential to drive forward the circular economy. While several corporations may have only just begun their journey towards greater circularity, with pilot projects confined to certain localities, the future up-scaling of socially inclusive recycling commitments by firms will be paramount in shifting the global economy from linear to circular, while addressing the imperative for decent work.

Corporate commitments to circularity sometimes explicitly aim to enhance the essential work performed by waste pickers, while tackling environmental degradation, resource depletion, or pollution. Yet sometimes, these commitments may be timely acts to avert stronger anti-plastic legislation, or a response to increasing calls to reform producer responsibility for managing the products and packaging they produce. Commitments made under such pressure may circumvent plastic taxes or protect corporate reputation, with commitments centred on materials, rather than people. Perhaps what sets the circular economy apart from sustainability is that while the latter offers a much broader holistic concept that treats the environment, the social, and the economic with equal weighting [13], the former prioritises economic systems and continued growth, with primary benefits for the environment and only implicit gains for social aspects [31]. In fact, Geissdoerfer et al. [31] attests that the main beneficiaries of the circular economy are the economic actors who implement the system, with private business playing a central role.

Finally, through exploring the long-considered importance of profit to multinational corporations [54], motivations driving these recycling commitments become more transparent. Individual companies stand to benefit financially from circularity due to reduced need for raw materials, waste avoidance [25], and the economic value retained in products after use to be reworked into new offerings [56], for example post-consumer recycled resins for new plastic products. There are therefore vested interests in the creation and implementation of corporate recycling commitments. Ultimately, the strong hierarchies we depict within plastic waste flows prevail (Fig. 2). And as materials are passed from waste pickers, to middle agents, to recycling plants, and to manufacturers, the value increases [101]. Those at the top of the plastic waste hierarchy benefit most. Large profits are won by industries manufacturing recycled resins for new products despite the low labour intensity, contrasting with the poorly paid but intensive work of waste picking [98]. Even with some new commitments and partnerships, at the base of the plastic waste recycling hierarchy, waste pickers have limited opportunity to add value to recyclable materials.

It is important to remember that while over 400 organisations have recently signed the New Plastics Economy Global Commitment to transform the plastic packaging sector [26, 27], the majority of companies globally have not yet employed recycling or circular economy schemes. Furthermore, signatories of this commitment include 200 businesses that are part of the plastic packaging value chain, yet make up only 20% of all plastic packaging used globally (*ibid.*). Financial viability is one reason for this discernible hesitation of many corporations to transition to more circular business models. The collection, sortation, and remanufacture process of recyclable materials often only breaks even or makes a loss, rendering recycling unattractive compared to using virgin materials [29]. This is especially true for plastics when the price of oil is low. Furthermore, high short-term capital costs may be borne from the initial implementation of post-consumer recycling practices since big investments are required to create radical change, presenting a risk of short-term financial loss. Thus, circular business models can often be more costly than their linear counterparts, especially in the early stages of the transition. To date, the number of corporate entities engaging with recycling schemes and more ambitious versions of circular economy remains rather small.

## The Missing Butterfly of Social Regeneration

The corporate protagonists of the circular economy are heavily reliant upon the work of waste pickers to achieve their ambitious goals. Furthermore, waste pickers, despite earning little and working in dangerous conditions, nevertheless depend upon this work to get by. This interdependence indirectly connects some of the least protected workers with global ambitions of major multinational companies via the flow of plastic into newly recycled packaging (see Fig. 2). A great deal of thought has gone into producing idealised models for circular flows, which emphasise the restoration and regeneration of materials through their collection, processing, repair, remanufacture, sorting, and recycling. Here, we propose a complementary model for social restoration and regeneration, emphasising some of the most disadvantaged workers involved with circular material flows. Our model highlights the need to act with care and responsibility towards people as well as the materials in these processes.

**Fig. 3:**
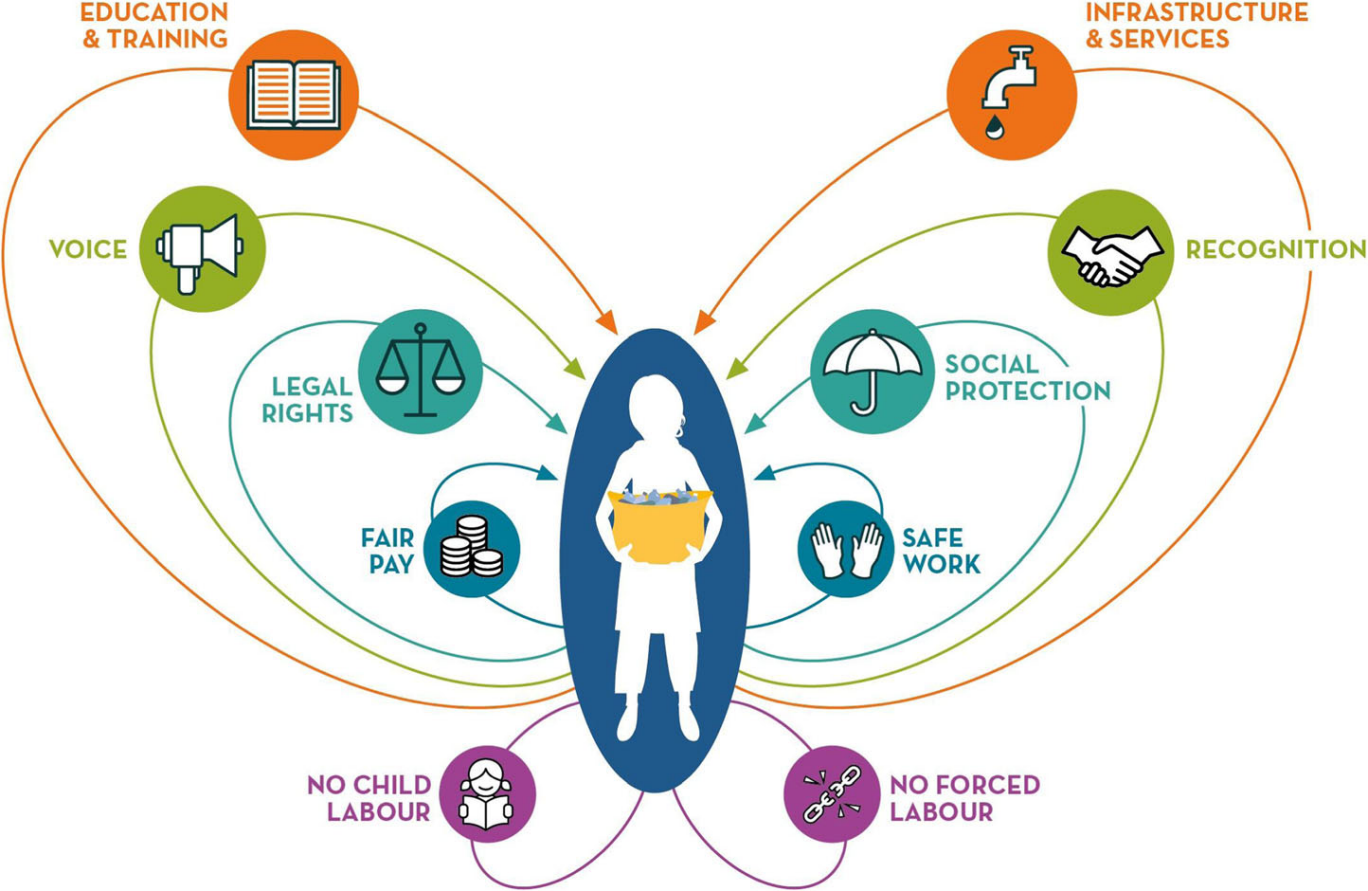
**A socially restorative butterfly for the circular economy**. Inspired by the Ellen MacArthur Foundation (EMF) [26, 27] circular economy system diagram infographic, which illustrates the technical and biological material flows through the ‘value circle’, and by the International Labour Organisation’s definition of Decent Work. The purple loops at the bottom show essential foundations of restorative work: no child or forced labour. The inner upper loops show the need for fair and safe conditions, in a wider context of legal rights and social protection, where workers are respected, and their voices are heard. The outer upper loops highlight the need for access to education, training, infrastructure, and services beyond work

The socially restorative butterfly (Fig. 3) mimics the circular economy system diagram, produced by the Ellen MacArthur Foundation (EMF) [26, 27] which with two sets of wing-like loops looks something like a butterfly. That original diagram shows two series of concentric loops, one technical and the other biological. The technical set of loops show materials being brought back into use, with the goal being to keep materials at their highest value for longer—thus, repair and maintenance take precedence over remanufacture, with recycling being the last resort to avoid landfill. This preference is shown by the inner loops showing higher priority goals. The other biological loop focuses on decomposition and regrowth of bio-based materials. To complement this intuitive visualization, we highlight the potential to restore and regenerate workers within the circular economy. While this has been developed through a study of waste pickers, this approach could be usefully applied to other workers in the circular economy and beyond.

There is a stark mismatch between the International Labour Organisation’s definition of decent work and the circumstances of many waste workers as described above. The decent work definition refers to job quality at multiple levels—the level of the individuals’ characteristics (are they a child, is this forced labour?), at the work environment (is it safe to work there?), and at an aggregate level (is there social protection?) ([69], p.177). Our model refers to these levels as well—the individuals and the context in which people are working, in terms of the work environment and the wider societal structures in which this work plays out. Like the Ellen MacArthur ‘butterfly’, the most pressing needs are the inner loops and the lowest loops, yet here all loops should be fulfilled to reduce vulnerability and precarity, which would directly contribute to meeting the Sustainable Development Goals on poverty, hunger, gender equality, decent work, inequality, and thoroughly responsible production. The key components outlined here amount to a call for fair trade recycled plastics supply chains.

Over the past decade, several cities have seen the organisation of waste pickers into cooperatives and associations, especially throughout Latin America. Gutberlet et al.’ [38] use of the combined discursive framework of the social and solidarity economy and ecological economy illuminates how community-based waste picking practices do not just offer a circular economy but provide sustainable livelihoods and decent working conditions. For example, unlike autonomous waste pickers, the organised waste picker cooperative in Argentina *Reciclando Sueños* (recycling dreams) signed a contract with the Limpex chemical company in July 2016 (*ibid.*), leading to a doubling of recycling rates and higher incomes for waste pickers, becoming a milestone in their struggle for recognition as a public service provider. This example flags some imperfections; there is a need for additional resources and partnerships, and these partnerships can leave waste picker organisations vulnerable to power asymmetries with their corporate partners, which can lead to moral issues [35]. These imperfections lead us to explore several approaches to ensuring better working conditions for waste pickers in order to foster both material and social regeneration at the individual, work environment, and aggregate levels.

The lower wings of the butterfly (Fig. 3) provide the foundation for restorative work, in that forced labour and child labour should be absent. The inner loops of the upper wings show ‘safe work’ and ‘fair pay’ as high priority, followed by the need for social protection and legal rights, then voice and recognition, in a context of access to education, training, infrastructure, and services. Initiatives targeted at combatting one element may also address others. Fairer pay has been achieved through the establishment of buy-back centres which work to mitigate volatile market prices for plastic waste and exploitation by middle agents, depicted in the third layer of the hierarchy (Fig. 2). Due to the dependence that waste pickers and collectors have on the middle agents, they are able to fix prices at low levels [85]. The for-profit company Mr Green Africa, located in Nairobi, Kenya, operates a business model where fixed prices at 19 Kenyan Shillings per kilogram of plastic are estimated to be 30% higher than prices offered by other local waste buyers [30]. This has not only addressed the inner-loop element of fairer pay by providing a guaranteed stable monthly income and prevented the possible mistreatment of waste pickers by middle agents who themselves may have lost work as a result. In this example, close interactions between waste pickers and formally employed staff have shifted negative stereotypes of informal waste workers (*ibid*.). This is significant given the long history of marginalisation and stigmatisation of waste pickers across the globe, despite their valuable contributions.

Governments must play an integral role in facilitating circular activity. This is especially true in the informal recycling sector. Various initiatives have been implemented in Latin America, Africa, and Asia intended to integrate and formalise waste pickers, to enhance their legal rights and social recognition. While not all are successful, targeted action can lead to social protection [93] and legal protection [37], higher wages and infrastructure provision [19], and engagement in formalised service contracts (Medina, 2008). Alongside government, other key actors and stakeholders initiating change include social enterprises, co-operatives, non-governmental organisations, and the companies from which plastic waste originates. Table 3 outlines some of the variety of socially regenerative initiatives adopted by diverse stakeholders involved in waste management which target the socially regenerative elements outlined in Fig. 3.

**Table 3:** Socially regenerative and restorative interventions with waste pickers. These examples demonstrate how a range of actors are well positioned to intervene, including waste pickers, NGOs, government, and businesses

Socially regenerative element	Mechanisms	Examples
Protecting children and forced labourers *To fix unacceptable practices*	National policy; NGO awareness campaigns	*Bolsa familia* Brazil—prevented exploitation of children because they were in school. >40,000 children have left waste picking and gone to school (Medina, 2008). Association for Rural and Urban Needy (Arun) and Save the Children India (STC) have facilitated school enrolment for children forced into waste-picking in India.
Safer working and living conditions *To fix dangerous livelihoods*	Sanitation reforms; Provision of essential equipment	Unilever South Africa to provide Personal Protective Equipment to waste pickers, and a proposal from the Minister of Environmental Affairs, Forestry and Fisheries to provide support through a National Solidarity Fund [70].
Pricing stability *To fix unpredictability*	Fixed pricing; Co-operatives; Fair-trade; Buy-back centres	Eco Brixs, Uganda, pays collectors a fixed price, giving advanced notice of price changes (Eco Brixs, 2021). Mr Green Africa in Kenya trades at a fixed price which is 30% more than middle agents offer [30].
Higher wages *To fix working poverty*	Government payment schemes; Corporate commitments	Municipal payment scheme Bogotá 2013, Columbia, remunerates registered waste pickers at 87,000 pesos/tonne recyclables collected, in addition to sale of material at market prices (Dias, 2016). Unilever’s 2021 global commitment to ensure living wages are paid by all of its suppliers of goods and services [6].
Social protection *To fix lack of healthcare and social safety net*	National policy; Universal health programmes	Government of Senegal’s universal health program enables low socio-economic groups to pool resources and access lower cost healthcare [93].
Worker rights and voice *To fix misrepresentation, exclusion and marginalisation*	National certifications; Inclusion of waste pickers in legislation; Legal protection	National Solid Waste Legislation, *Política Nacional de Resíduos Sólidos*, Brazil: Law 12.305/10, allows waste pickers to engage in service contracts with the city [37]. July 2017 Waste Pickers’ Protest in Johannesburg, South Africa, against the Separation at Source program to privatise waste management led to development framework for waste picker integration (Pillay, 2017)
Formalisation *To fix informality*	Public-Private Partnerships; Buy-back centres; Formal contracts	Bogotá Association of Waste Pickers: Municipality provide infrastructure/equipment and pickers provide the labour (Dias, 2006) Belo Horizonte, Brazil integrated waste picker cooperatives into municipal waste management systems (Medina, 2008).
Gender equality measures *To fix exploitation and gender inequality*	Co-operatives; Public-Private Partnerships	Solid Waste Collection and Handling (SWACH) in Pune, India: 80% of cooperative’s workers are women who benefit from a pro-poor Public Private Partnerships [52].
Social recognition & respect *To fix the stigmatization of waste pickers*	Provision of uniforms; Public campaigns	Waste pickers of four cooperatives in Santiago de Chile receive green uniforms and a municipal identification card to help with their institutionalisation. Successful in building trust in higher-income neighbourhoods and encouraging their general acceptance by society [65].

We have already explored how multinational corporations and their increasing adoption of recycling commitments hold the potential for a future of plastics that is not just circular, but socially regenerative for the least advantaged people in their supply chain. Reviewing the problem and possible responses, we call for an industry-wide shift to fair trade recycled plastic as one component of circularity. Fair trade comprises the social and solidarity economy, offerings an alternative approach to largely unethical mainstream modes of production and consumption. However, fair trade does not have to create a whole new system in radical response to problems in the basic nature of global capitalism as some versions argue [40]. It can also promote more inclusive and equitable trade within existing structures, for example between the differing levels represented in the plastic waste hierarchy (Fig. 2). We recognise the efforts of the global partnership between Plastics for Change and The Body Shop in driving this forward [71] who have been certified by the World Fair Trade Organisation. The principles of fair trade align with many of the elements of the socially restorative butterfly we propose here. Therefore, by integrating fair trade recycled plastic into theories and practices of the circular economy, responsible supply chains that deliver economic, environmental, and social benefits can be made a reality. Ultimately, narrowing, closing, and slowing loops are imperative, and requires ambition beyond recycling. What will define a truly transformational circular economy will be a parallel emphasis on restorative, regenerative, and enabling social dimensions.

## Conclusion

This paper responds to Rathore’s call to think not only of material flows but about circulating bodies and labour, of the ‘assemblage of people, places and material and how they interact and intersect to create value’ ([74], 182). We highlight how the quality of work that accompanies the recycling loops within circular processes in many low- and middle-income countries remains a blind spot in much of the circular economy literature, policy, and practices. It is already established that waste work maps onto existing inequalities, offering a meagre livelihood for some of the most marginalised groups [11], and this paper shows how poor-quality jobs in unsafe conditions paid at poverty wages without a social safety net recreate and regenerate these injustices. While idealised models of the circular economy prioritise reuse, repair, and remanufacture over recycling, recycling remains a popular first step towards circularity. Given this, circular economy thinkers and practitioners must pay attention to the working conditions for those engaged in recycling loops.

We have presented evidence of the critical role of waste pickers in the recycling loop of the circular economy, demonstrating how across countries they handle large proportions of all recycled materials. In the context of increasingly ambitious corporate commitments to the improved management of plastic waste, waste picking work is set to increase. While interventions at the local and national scales have sought to improve the circumstances of waste pickers through mechanisms such as fixed pricing, government contracts, and sanitation programmes, the work of most waste pickers continues to be precarious and dangerous. As the circular economy pushes ahead with new research and technological innovation, we must attend to the social and economic impacts of existing circular practices. At present, active responsibility is much more progressed for plastics than for the workers handling the waste. Perhaps, this is in part because branded packaging signals corporate responsibility for actively adding plastics to the environment, whereas informal work several steps removed from a corporate entity *appears* more morally disconnected. The themes of this desk-based review could be extended through primary research into (1) the experiences, solutions, and aspirations of the people doing waste picking work, and (2) the organisations already adopting more socially restorative versions of waste collection.

Given how new circular models are being purposefully designed and built, it is essential that sometimes-invisible workers are intentionally engaged in this process to achieve an inclusive circular economy. ‘*Our common future is not already mapped out; it is still to be won*’ [20]. Thus, we propose a new model of social regeneration for waste pickers in the circular economy: a socially regenerative butterfly for the circular economy (Fig. 3). Building upon this, we recommend a new industry-wide standard of fair trade recycled materials.

## Data Availability

The data and material presented here are all sourced from the public domain and are referenced accordingly.
